# Assessment and Management of Sleep Disturbance in Cirrhosis

**DOI:** 10.1007/s11901-018-0390-1

**Published:** 2018-02-13

**Authors:** Chiara Formentin, Maria Garrido, Sara Montagnese

**Affiliations:** 1 Department of Medicine, University of Padua, Via Giustiniani, 2, 35128 Padua, Italy; 2 Department of Physiology, Neuroimmunophysiology and Chrononutrition Research Group, Faculty of Science, Avda. Elvas s/n, 06006 Badajoz, Spain

**Keywords:** Sleep, Insomnia, Circadian rhythms, Hepatic encephalopathy

## Abstract

**Purpose of Review:**

This review presents an in-depth overview of the sleep–wake phenotype of patients with cirrhosis, together with available pharmacological and non-pharmacological treatment strategies. A set of simple, practical recommendations is also provided.

**Recent Findings:**

The understanding of the pathophysiology of sleep disorders in this patient population has improved over the past decade, especially in relation to the interplay between homeostatic and circadian sleep regulation. In addition, new tools have been utilised for both screening and in-depth investigation of the sleep–wake profile of these patients. Finally, a number of studies have evaluated the efficacy of novel treatment strategies, often with encouraging results.

**Summary:**

Since sleep disturbances are common in patients with cirrhosis, more so than in patients with other chronic diseases of similar severity, their assessment should become routine hepatological practice, along with the initiation of adequate treatment.

## Introduction

Sleep disturbances are common in patients suffering from liver cirrhosis [1–3] and they impinge on their health-related quality of life (H-RQoL) [4], thus representing a challenging topic of clinical relevance.

Difficulties sleeping can be due to several factors: pain and discomfort associated with the disease, poor sleep hygiene, medications that interfere with sleep, pruritus and fatigue (especially in primary biliary cirrhosis) [5], but at the same time they are pathophysiologically related to liver disease, and they represent, at least to a certain extent, a direct consequence of liver dysfunction. Notably, insomnia is also reported in well-compensated cirrhotics, with no evident reasons for disturbed sleep such as itching, tense ascites, or treatment with diuretics [1, 4].

The recognition of sleep disturbance in cirrhosis and the understanding of its underlying pathophysiological mechanisms are of crucial importance in the management of the disease, since they may translate into potential improvement of these patients’ quality of life.

## Phenotype

Sleep disturbances in cirrhosis were first characterized in 1954, when Sherlock et al. described sleep–wake inversion (i.e. the combination of restless nights and excessive daytime sleepiness) in patients with severe overt hepatic encephalopathy (HE) [3]. These features represent an extreme of the wide range of sleep disturbances exhibited by cirrhotic patients, even by those without signs of overt HE.

Few studies are available regarding the prevalence of sleep disturbance in cirrhotic patients without overt HE [1, 2, 6•, 7]. These document a prevalence of difficulties falling asleep, multiple night awakenings and daytime sleepiness varying from 27 to 70% [1, 2, 4, 6•, 7, 8].

A list of the most common sleep disorders described in patients with liver cirrhosis, divided into three groups from an aetiological point of view, follows [1–4, 6•, 9••, 10–16]:

A. Sleep–wake signs/symptoms associated with cirrhosis:
  prolonged sleep latency/difficulty with sleep initiation/sleep onset insomnia;
  frequent nocturnal awakening/fragmented sleep/difficulty with sleep continuity/sleep maintenance insomnia;
  difficulty falling asleep after nocturnal awakenings;
  shortened sleep duration;
  poor sleep quality/reduced ability to produce restorative sleep/unrefreshing sleep.
B. Sleep–wake signs/symptoms secondary to the aetiology of liver disease:
  unusual events associated with sleep, such as heavy snoring, apnoea, or periodic limb movements (metabolic cirrhosis);
  delayed sleep habits (delayed sleep onset, delayed wake-up time), preference for evening activities and insomnia (alcohol-related cirrhosis) [17–19].
C. Sleep–wake signs/symptoms related to HE:
  hypersomnia/somnolence/excessive and inappropriately timed sleepiness/numerous and long daytime naps (up to sleep–wake inversion in severe overt HE).

A summary of the available studies, where phenotypic characteristics of sleep disturbances in cirrhotic patients are described, is provided in Table 1.

**Table 1:** Studies describing the sleep–wake phenotype of patients with cirrhosis

First author	Year	Title *Journal*	Patients and methods	Results	Conclusions and notes
Sherlock S	1954	Portal-systemic encephalopathy; neurological complications of liver disease *Lancet*	18 patients with liver disease; blood ammonium and EEG. Nervous disorders induced by the administration of nitrogenous substances.	Importance of three clinical factors: portal-systemic venous collaterals, hepatocellular disease, nitrogenous substances in the intestine.	Nitrogenous substances of portal venous origin, normally metabolised in the liver, may reach the systemic circulation through a damaged liver, through portal collateral channels, or through both, and cause portal-systemic encephalopathy.
Kurtz D	1972	Night sleep in porto-caval encephalopathy *Electroencephalography and Clinical Neurophysiology*	18 polygraphic recordings from 15 cirrhotics. EEG and behavioural aspects of sleep, general characteristics of sleep, vegetative changes and cyclic organization of sleep during the night.	Correlations between clinical and EEG severity + arterial ammonia level and EEG features, duration of sleep and its organization.	The conservation or not of the physiological character of night sleep is a function of the state of vigilance and the diurnal disturbances of the EEG. With worsening of HE, pathological variations of physiological sleep appear.
Steindl PE	1995	Disruption of the diurnal rhythm of plasma melatonin in cirrhosis *Annals of Internal Medicine*	7 patients with cirrhosis and 7 controls. Neuropsychological testing and sleep diaries. Plasma melatonin levels measured every 30 min for 24 h by radioimmunoassay.	Elevated melatonin levels during daytime hours in cirrhosis; the time of onset of melatonin increase and the time at which melatonin levels peaked, significantly delayed. More nocturnal awakenings and more frequent daytime naps in cirrhotics.	Disruption of the diurnal rhythm of melatonin may reflect alterations of circadian function that could contribute to the disturbances of the sleep–wake cycle in cirrhosis.
Córdoba J	1998	High prevalence of sleep disturbance in cirrhosis *Hepatology*	Sleep questionnaire (*n* = 44) and actigraphy (*n* = 20), results compared with those of subjects with CKD and healthy controls; presence of subclinical HE, chronotype, and individual’s affective state also analysed.	Unsatisfactory sleep in cirrhosis and CKD vs healthy controls. Sleep disturbance in cirrhosis not associated with clinical parameters nor with cognitive impairment. Unsatisfactory sleep associated with higher scores for depression and anxiety. Unsatisfactory sleep in cirrhosis associated with delayed bedtime, delayed wake-up time, and evening chronotype.	Sleep disturbance frequent in cirrhotic patients without HE, maybe related to abnormalities of the circadian timekeeping system.
Mostacci B	2008	Sleep disturbance and daytime sleepiness in patients with cirrhosis: a case control study *Neurological Sciences*	178 cirrhotics compared to a control group. Sleep features and excessive daytime sleepiness evaluated by the BNSQ and the ESS.	Complaints of more daytime sleepiness, sleeping badly at least three times a week, difficulties falling asleep and frequent nocturnal awakening in cirrhosis.	Confirm of high prevalence of sleep disturbance in cirrhosis, insomnia and daytime sleepiness as main complaints.
Montagnese S	2009	Sleep and circadian abnormalities in patients with cirrhosis: features of delayed sleep phase syndrome? *Metabolic Brain Disease*	Sleep monitored for 2 weeks, at home, with sleep diaries and actigraphy, in 35 patients with cirrhosis and 12 healthy controls; aMT6s measured over 56 h, to assess circadian rhythmicity.	Later wake up and get up times, with a more fragmented sleep in patients. Significant 24-h urinary aMT6s rhythms observed in 26 of 33 patients; 20 patients had a normally timed urinary aMT6s peak, while it was delayed (> or = 06:00) in the remainder. Significant correlations between abnormalities in the urinary aMT6s profile and indices of sleep timing; parallel delays in sleep habits and urinary aMT6s peaks.	Delayed circadian rhythms and delayed sleep habits is reminiscent of ‘delayed sleep phase syndrome’; condition managed by attempting to resynchronise the circadian clock by exposure to bright light shortly after morning awakening.
Montagnese S	2009	Night-time sleep disturbance does not correlate with neuropsychiatric impairment in patients with cirrhosis *Liver International*	87 patients, classified as unimpaired or minimal/overt HE. 19 healthy volunteers as controls. PSQI and ESS. 36-item short form health profile (SF-36v1) and the chronic liver disease questionnaire to assess H-RQoL.	Patients slept significantly less well than the healthy volunteers and had more pronounced daytime sleepiness. No significant relationships between sleep indices and the presence/degree of HE. Night-time sleep disturbance as independent predictor of poor H-RQoL.	Sleep–wake abnormalities are common in cirrhosis; they significantly affect H-RQoL but are not related to the presence of HE.
Llansola M	2012	Progressive reduction of sleep time and quality in rats with hepatic encephalopathy caused by portacaval shunts *Neuroscience*	Rats subjected to PCS to induce HE. Another group of rats fed with an ammonium-containing diet to induce hyperammonemia. Polysomnographic recordings acquired for 24 h in control, PCS, and hyperammonemic rats at 4, 7 and 11 weeks after surgery or diet.	PCS rats show a significant reduction in REM and NREM sleep time and increased sleep fragmentation. Hyperammonemic rats show decreased REM sleep, with no changes in NREM sleep or sleep fragmentation.	PCS rats are a good model to study sleep alterations in HE, their mechanisms, and potential treatment. Mild hyperammonemia mainly impacts mechanisms involved in REM generation and/or maintenance but does not seem to be involved in sleep fragmentation.
Samanta J	2013	Correlation between degree and quality of sleep disturbance and the level of neuropsychiatric impairment in patients with liver cirrhosis *Metabolic Brain Disease*	On the basis of PHES, 100 cirrhotics divided into those having MHE and those not. Sleep disturbance measured with PSQI and ESS and HRQOL with SF-36(v2) questionnaire.	60 patients were ‘poor sleepers’ while 38 had excessive daytime sleepiness. Significant correlation between PHES, PSQI and ESS, independently strong correlation between poor cognition and sleep disturbance and excessive daytime sleepiness in cirrhosis. Significant correlation between PSQI and ESS and scores of SF-36(v2).	Night time sleep disturbance and excessive daytime sleepiness have significant relation with the neuropsychiatric impairment in patients of cirrhosis and are significantly associated with the observed impairment in HRQOL.
Montagnese S	2013	Sleep–wake profiles in patients with primary biliary cirrhosis *Liver International*	74 patients with PBC, 79 healthy volunteers and 60 patients with cirrhosis: formal assessment of sleep quality/timing, diurnal preference and daytime sleepiness. Assessment of fatigue, quality of life and the daytime course of sleepiness/pruritus in PBC.	Sleep timing significantly delayed in both patients with PBC and cirrhosis, compared to healthy volunteers. In PBC, delayed sleep timing associated with impaired sleep quality. Physiological daily rhythm in sleepiness, with early afternoon/evening peaks. Prolonged sleep latency and earlier wake-up times in patients with PBC and significant pruritus. Significant correlations observed between sleep timing and quality of life.	Delay in sleep timing associated with impaired sleep quality/quality of life in PBC. In addition, an interplay observed between diurnal preference and the daytime course of pruritus/sleepiness.
Teodoro VV	2013	Polysomnographic sleep aspects in liver cirrhosis: A case control study *World Journal of Gastroenterology*	42 cirrhotics and 42 controls: questionnaire about habits, behaviours, and complaints related to sleep and polysomnography. Sleep parameters compared between the two groups, and separate analyses performed among classes of Child-Pugh classification in the cirrhotic group.	Cirrhotic group: lower sleep efficiency, increased latency, lower percentage of REM sleep and higher frequency of periodic limb movements. Significant reduction of REM sleep stage occurrence in individuals with severe liver disease (Child C patients) compared to Child A/B.	Cirrhosis is associated with shorter sleep time, reduced sleep efficiency, increased sleep latency, increased REM latency and reduced REM sleep. Additionally, disease severity influences sleep parameters.
Gencdal G	2014	Sleep disorders in cirrhotics; How can we detect? *Liver International*	131 cirrhotic patients - age-matched healthy volunteers. Both groups completed PSQI and STSQS.	131 cirrhotics and 18 volunteers. Sleep disorders in cirrhotics and control group: 56.5 and 27.8% by PSQI, 49.6 and 16.7% by STSQS. More frequent sleep disorders in decompensated than compensated cirrhosis.	Sleep disorders are common in cirrhotics and STSQS could be an appropriate and practical method for diagnosis of sleep disorders in these patients. We can use it in cirrhotic patients at outpatient clinics.
AL-Jahdali H	2014	Prevalence of insomnia and sleep patterns among liver cirrhosis patients *Journal of Circadian Rhythms*	200 patients with cirrhosis, ICSD-2 definition to assess the prevalence of insomnia, information about sleep patterns, demographic data, cause and severity (Child-Pugh) of cirrhosis.	Significant association between cirrhotic patients without OHE and sleep disturbances. Insomnia, delayed-phase sleep and excessive daytime sleepiness common among cirrhosis.	High prevalence of insomnia in patients with liver cirrhosis.
Saleh K	2017	Sleep in ambulatory patients with stable cirrhosis of the liver *Sleep Medicine*	Patients with end-stage liver disease (13) or severe HF (24). Full-night, attended polysomnography along with laboratory tests.	Severe PLMS and excessive arousals in cirrhosis compared to HF. Significant correlations between severity of PLMS vs blood levels of bilirubin and ammonia.	First polysomnographic study of patients with stable cirrhosis demonstrating severely disturbed sleep infrastructure and presence of excessive PLMS with arousals. These polysomnographic findings confirmed the subjective reports of poor sleep by patients with cirrhosis.

*aMT6s*, urinary 6-sulphatoxymelatonin; *BDI*, Beck Depression Inventory; *BNSQ*, Basic Nordic Sleep Questionnaire; *CKD*, chronic kidney disease; *CLD*, chronic liver disease; *EEG*, electroencephalogram; *ESS*, Epworth Sleepiness Scale; *HE*, hepatic encephalopathy; *OHE*, overt hepatic encephalopathy; *HF*, heart failure; *H-RQoL*, health-related quality of life; *NAFLD*, non-alcoholic fatty liver disease; *PBC*, primary biliary cirrhosis; *PCS*, portacaval shunt; *PGWBI*, Psychological General Well-Being Index; *PLMS*, periodic limb movements during sleep; *PSQI*, Pittsburgh Sleep Quality Index; *REM*, rapid eye movements; *NREM*, non-rapid eye movements; *STSQS*, Sleep Timing and Sleep Quality Screening questionnaire

## Pathophysiology and Relation to Hepatic Encephalopathy

The origin of sleep–wake disturbances in cirrhosis is likely to be multifactorial.

It has been observed that patients with cirrhosis present delayed sleep habits/evening preference [1]. These features are, in turn, associated with impaired sleep quality. Evening type cirrhotic patients present an increased sleep latency and worse sleep quality than their counterparts with earlier sleep times [4].

Based on the assumption that sleep is regulated by two primary components, a homeostatic process, that builds up during wakefulness and declines during sleep [20], and a circadian process, with near 24-h periodicity, Montagnese et al. [9•] have proposed a model to explain the abnormal interaction between homeostatic and circadian components of sleep in cirrhotic patients.

With regard to the homeostatic sleep–wake regulation in patients with cirrhosis, it has subsequently been shown that hyperammonaemia/HE correlates with daytime sleepiness and disruption of sleep architecture. In 2012, Bersagliere et al. [21] demonstrated that the administration of a so-called amino acid challenge (AAC), a mixture of 54 g of amino acids which is used to simulate hyperammonaemia/mild HE, leads to a significant increase in daytime subjective sleepiness and changes in the sleep EEG architecture in cirrhotic patients, with a consequent impairment in their ability to generate restful sleep. These findings support the interpretation of HE as a vigilance defect [4]. Indeed, the absence of daytime sleepiness has been shown to have a high negative predictive value in relation to the occurrence of HE-related hospitalizations, thus suggesting that patients who do not report daytime sleepiness may not need formal neuropsychiatric assessment or particularly close monitoring in relation to their risk of HE [22].

Based on the hypothesis that the sleepiness-inducing effect of ammonium is mediated by adenosine, a known regulator of sleep/wake homeostasis, a recent study performed on hyperammonaemic rats (i.e. fed with an ammonium-enriched diet for 4 weeks) showed, after sleep deprivation, a larger increase in adenosine levels in hyperammonaemic animals in comparison to controls, highlighting the role of adenosine in mediating the sleepiness/sleep-inducing effects of hyperammonaemia [23••]. Interestingly, the increase in putrescine, a molecule that is presumably correlated with neuroinflammation [24] and/or blood brain barrier alterations [25], was higher in the hyperammonaemic group, pointing to a more than additive effect underlying the association between hyperammonaemia/neurocognitive impairment and insomnia.

However, there are multiple, mostly unexplored mechanisms through which cirrhosis and its complications might affect sleep–wake patterns. Further pathophysiological factors may contribute to sleep impairment, as sleep abnormalities have also been reported in patients with little or no evidence of neuropsychiatric disturbance related to HE [2] and in a number of studies no significant relationships have been observed between night sleep disturbances and the presence/degree of HE [4, 22].

Abnormalities in the circadian rhythm of melatonin of both central (reduced cerebral sensitivity to dark/light cues as a consequence of a dysfunction of the central suprachiasmatic nucleus circadian clock) and peripheral origin (reduced melatonin clearance, high daytime melatonin levels and low urinary 6-sulfatoxymelatonin concentration) may play a role, although without offering a comprehensive explanation for the observed sleep–wake disorders [11, 26].

Disturbances in the 24-h rhythm of skin temperature have been recently reported in patients with cirrhosis [27]. In physiological conditions, skin temperature starts to decrease in the evening and a redistribution of heat from core to peripheral regions, driven by vasodilatation of distal skin sites, occurs [28–30]. The peripheral heat loss is measured by the distal-proximal gradient (DPG), which is an excellent predictor of sleep latency [28, 29]. In patients with cirrhosis, an impaired thermoregulation, in terms of absolute values and time-course of DPG, has been reported [27]. These alterations may be explained by the hyperdynamic circulatory syndrome [31], caused by the generalized state of vasodilatation, which may hamper heat dissipation in these patients. Moreover, such abnormalities were shown to parallel the severity of the disease and the associated sleep–wake abnormalities [27].

**Fig. 1:**
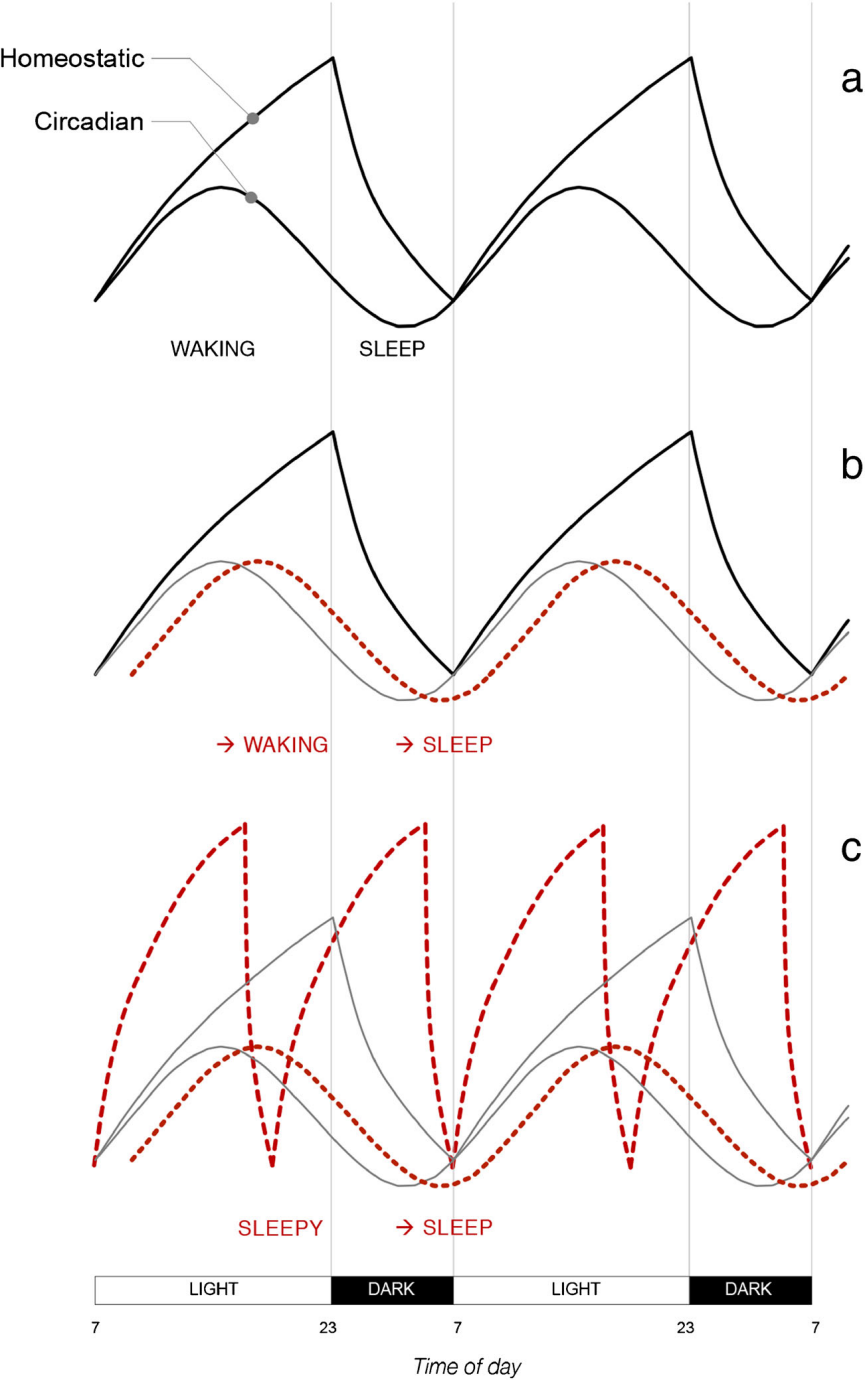
Cirrhosis-associated abnormalities within the context the two-process model of sleep regulation (*adapted and updated from* Fig. 1 *of Reference* 9). (a) Normal interaction between the circadian oscillation in sleep propensity and the increase in homeostatic sleep pressure during the waking hours: the greater the distance between the two curves (23:00), the higher the sleep propensity (adapted from [20]). (b) Abnormal interaction between the homeostatic regulation (black line) and the delayed circadian rhythm (red dotted line) in a patient with cirrhosis; gray line: reference circadian oscillation in the healthy population. The lack of synchrony between the two processes leads to a jet-lag East-type sleep disorder, which could contribute to the observed difficulties in commencing (increased latency) and maintaining sleep (fragmentation) (adapted from [9•]). (c) Abnormal interaction between homeostatic fluctuations (red broken line) and shifted/delayed circadian rhythm (red dotted line) in a patient with cirrhosis and HE; gray lines: reference circadian oscillation and homeostatic build-up in the healthy population. Hyperammonaemia/HE results in magnified and short-lived adenosine responses to the build-up of sleep pressure during the waking hours. This translates into an inability to generate slow-wave, restorative sleep and in a less efficient recovery from sleep deprivation.

A model for the interaction and the effects of circadian and homeostatic dysfunction in cirrhosis, based on the current available evidence, is presented in Fig. 1 [20].

## Comorbidities

The presence of comorbid conditions should be considered in the assessment of sleep impairment in cirrhotic patients and it represents a non-negligible issue in the evaluation and proper treatment.

### Obstructive Sleep Apnoea

An exemplifying condition is represented by obstructive sleep apnoea (OSA) in metabolic cirrhosis. The prevalence of this well-recognized cause of sleep fragmentation and excessive daytime somnolence [32] is increased in obese patients [33]. Emerging data support the hypothesis that OSA-related oxidative stress and hepatic ischemia-reperfusion injury may contribute to the progression from steatosis to nonalcoholic steatohepatitis (NASH) and, ultimately, metabolic cirrhosis [34–38].

Bajaj et al. [39] analysed the complex relationship between OSA and metabolic cirrhosis in terms of sleep quality, daytime sleepiness, cognition and driving simulation, highlighting the fact that OSA is a cause of daytime sleepiness in cirrhosis and concluding that OSA should be considered as a modulator of cognitive function and sleep quality in chronic liver disease. Moreover, Continuous Positive Airway Pressure (CPAP) has been shown to be effective in the treatment of OSA both in patients with and without cirrhosis, significantly improving executive function and sleep quality in both groups [39].

### Alcohol Misuse

The negative influence of alcohol consumption on the sleep–wake cycle represents another example of the complex relationship between liver disease and its pathogenetic factors.

In recent years, a better understanding of the relationship between sleep homeostasis and genetic substrates of circadian physiology on one hand, and psychiatric disorders, including alcohol misuse, on the other, has lent support to the idea that alcohol consumption and chronobiological disruption reciprocally interact, thus resulting in a vicious cycle [40, 41]. Indeed, reciprocal interactions occur between the circadian system and ethanol consumption at both physiological and genetic levels [42]. Studies on animal models suggest that mutations of circadian clock genes (e.g. Per2 and CLOCK), are associated with increased alcohol intake, which in turn is promoted by perturbed circadian behaviour [43].

In addition, the adverse effects of alcohol consumption on sleep are well characterised both in healthy individuals and alcohol misusers. In social drinkers, acute alcohol intake determines a reduced sleep onset latency and an increased quality (delta power) and quantity of NREM sleep [44, 45], whereas severe insomnia, excessive daytime sleepiness, and altered sleep architecture are the consequences of prolonged alcohol misuse [46, 47]. Abstinence from alcohol in alcohol misusers results in severe and protracted sleep disruption, with insomnia, sleep fragmentation, and alterations in sleep architecture, that may persist for several years after withdrawal. Furthermore, sleep impairment is a predictive factor of relapse [45].

Therefore, it is reasonable to hypothesize that circadian deregulation (i.e. delayed sleep habits, irregular life-style, lack of sleep-wake, food intake and physical activity routines) may promote alcohol misuse, which in turn disrupts sleep and alters circadian rhythms.

### Primary Biliary Cirrhosis

Sleep disorders complained by patients with primary biliary cirrhosis (PBC), a chronic autoimmune cholestatic disorder, may be partially explained by symptoms of liver disease, notably fatigue and pruritus [48, 49].

A significant correlation has been observed between the severity of pruritus and sleep impairment. PBC patients who show the greatest perception of pruritus and significantly complain about itching present longer sleep latency and earlier wake-up times, when compared to patients who are not considerably troubled by this symptom [5].

Similarly, fatigue negatively influences sleep–wake habits in PBC patients as it is significantly correlated with sleepiness in the morning and early afternoon hours [5, 48].

### Hepatitis C Virus Infection

It has been observed that over half of patients with chronic hepatitis C complain of chronic fatigue, depression, reduced quality of life and sleep disturbance, in particular daytime sleepiness and poor sleep quality [50, 51]. The combination of such signs and symptoms has been termed “hepatitis C virus (HCV) syndrome” [52, 53] and it includes both hepatic and extra-hepatic manifestations of the infection, which are largely independent of the stage of liver fibrosis and the genotype [54].

In a study conducted on HCV-infected patients, with the aim of characterizing sleep disturbances in this population by means of actigraphy, questionnaires and sleep diaries, patients achieved lower quality of life scores and higher scores for depression, fatigue and sleep disturbances, in particular higher nocturnal activity and worse sleep efficiency, than healthy controls. Fatigue and quality of life scores correlated with bad sleep quality and daytime sleepiness [55].

While the above serve as examples of how the aetiology of liver disease and related comorbidities may impinge on the ultimate sleep–wake profile of a patient with cirrhosis, the consideration of aetiology/comorbidities and the formal diagnosis/exclusion of concomitant sleep–wake disorders are recommended (vide infra).

## Tools

Sleep health can be assessed by measuring the following parameters:

- night sleep quality,
- sleep–wake timing,
- daytime sleepiness.

The evaluation of sleep–wake behaviour in patients with cirrhosis comprises a heterogeneous mix of methodologies which can be distinguished into subjective and objective/semi-quantitative.

### Subjective Sleep Assessment

This is based on daily sleep diaries and questionnaires. Sleep diaries have been widely utilized for collecting patient’s habitual daily routines over time in sleep–wake research. These diaries have been commonly adapted to the population/features of the study but a comparison between studies is difficult because of the absence of standardization. Recently, a ‘Consensus Sleep Diary’ with standard indices to evaluate sleep–wake disturbances and particularly insomnia has been approved [56]. There is agreement that such tool should provide information on a set of parameters including night sleep onset latency (SOL), wakefulness after initial sleep onset (WASO), total sleep time (TST), total time spent in bed (TIB), sleep efficiency, and sleep quality or satisfaction, which reflects a subjective global appraisal of each night’s sleep [56]. Based on this approach, some authors have utilized this standardized diary for evaluating self-perception of sleep quality in patients with cirrhosis, confirming delayed sleep-wake timing and frequent night awakenings [27].

The Pittsburgh Sleep Quality Index (PSQI) is the gold standard test among self-administered tools utilized for evaluating the subjective sleep quality and sleep disturbances over the preceding month, as well as for distinguishing between ‘good’ and ‘poor’ sleepers [57]. It consists of 19 individual items grouped in seven components: sleep quality, sleep latency, sleep duration, sleep efficiency, sleep disturbances, sleep medication, and daytime dysfunction, each component being weighted equally on a 0–3 scale. The sum of all components provides a total PSQI score (ranged between 0 and 21), where scores ≥ 5 identifies poor sleepers. The PSQI takes around 10 min to be filled in and 5 min to be scored. Depending on the severity of cirrhosis, time required to complete the test and answering properly all the items can become complicated. In these settings, using simplified and quick the questionnaires, such as the validated Sleep Timing and Sleep Quality Screening Questionnaire (STSQS) [58], becomes a more suitable option for the diagnosis of subjective sleep disturbances in this population. This questionnaire takes approximately 2 min to be completed, without extra time for scoring. STSQS collects information of sleep quality rated on a 1–9 analogue scale (1: ‘best sleep ever’, 9: ‘worst sleep ever’) providing also information about habitual sleep timing: bedtime, sleep latency, night awakenings, and wake-up/get-up time. Recently, Gencdal et al. [15] have confirmed a significant correlation between STSQS and PSQI for the diagnosis of sleep disturbances in patients with cirrhosis.

Excessive daytime somnolence is one of the manifestations of the abnormal sleep–wake rhythm in patients with cirrhosis [9•] and it is commonly evaluated by means of The Epworth Sleepiness Scale (ESS) [59]. Subjects rate their likelihood of ‘dozing off’ in eight different daytime situations: sitting and reading, watching TV, sitting inactive in a public place like a theatre or meeting, as a passenger in a car for an hour without a break, lying down to rest in the afternoon when circumstances permit, sitting and talking to someone, sitting quietly after lunch without alcohol, in a car, while stopped for a few minutes in traffic. The likelihood of ‘dozing off’ is rated from 0 (unlikely) to 3 (very likely). The higher the ESS score (range 0–24), the sleepier the subject. A score ≥ 11 is considered abnormal. The detection of a strong correlation between poor cognition and excessive daytime sleepiness [6•] highlights the importance of sleepiness evaluation in the analysis of sleep disturbances in this population.

### Objective Sleep Assessment

Although the assessment of subjective sleep quality provides valuable information and it is recommended for screening purposes, these tools do not offer quantitative information about sleep architecture or sleep stages.

For in-depth studies, polysomnography (PSG) represents the gold standard diagnostic tool. This technique monitors brain electrogenesis, eye movements, skeletal muscle activity, blood oxygen levels, and heart/breathing rhythms during sleep.

A small number of PSG studies of patients with cirrhosis are available [60–62].

A correlation between the severity of HE on one hand and PSG patterns, the duration of sleep and its organization on the other was first identified in 1972 by Kurtz et al. [63] who analysed 18 polygraphic recordings from 15 cirrhotics in different stages of encephalopathy [63].

In more recent years, polysomnographic features and sleep aspects in cirrhotic patients have been extensively characterized, as well as the role of liver dysfunction severity [60–62]. Teodoro et al. [60] have showed that cirrhosis is associated with shorter sleep time, reduced sleep efficiency, increased sleep latency, increased rapid eye movement (REM) latency and reduced REM sleep [60], confirming the existence of a disturbed sleep structure in this population.

Nevertheless, PSG is an expensive and labour-intensive tool. In addition, since it initially disrupts sleep, its application normally requires an adaptation night (i.e. first night polysomnography on for the subject to get used to it, second night actual recording) to avoid the well-known ‘first-night effect’ [64]. Due to the aforementioned limitations, this technique is generally employed in the research field and currently not included in the routine assessment of sleep quality.

Actigraphy is a semi-quantitative technique that records temporal rest-activity patterns and allows the analysis of micro/macrostructure of nocturnal sleep [65]. Moreover, it is cost-effective compared to PSG, unobtrusive and can be utilized in free-living conditions. The actigraph is a wrist-watch like device that includes an accelerometer to monitor the subject’s movements. The simple assumption underlying the technique is wake = movement; sleep = lack of movement. Recorded data are analyzed with a software package to estimate sleep parameters such as total time in bed, sleep latency, real sleep time, assumed sleep, wake time after sleep onset, or number of awakenings per night. In liver units, actigraphy has been used in combination with complementary structured interviews or validated questionnaires with the idea of obtaining more detailed information about sleep–wake cycle beyond data provided by body movements, confirming poor sleep quality, prolonged sleep latency and reduced sleep efficiency in cirrhotic patients [66•, 67].

**Fig. 2:**
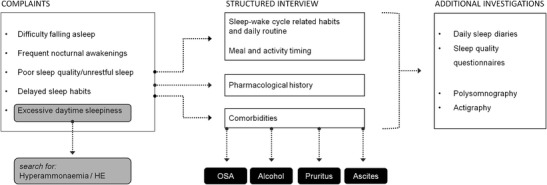
Practical recommendations for the assessment and management of sleep–wake disturbance in patients with cirrhosis

A list of the most important steps that should not be neglected in the routine assessment and management of sleep disorders in cirrhotic patients, including practical recommendations and diagnostic advice provided by the literature of the last few years, is reported below and summarized in the algorithm shown in Fig. 2.

- Formal diagnosis/exclusion of concomitant sleep–wake disorders that may be responsible for/contribute to sleep impairment.
- The presence of comorbidities/concomitant factors, that may contribute to sleep impairment (i.e. OSA, alcohol, pruritus in PBC, ascites), should be sought for and a proper treatment should be initiated [68].
- Specific enquiry for sleep–wake disturbances and detailed characterization of symptoms (excessive daytime sleepiness, difficulty falling asleep, frequent nocturnal awakenings/fragmented sleep, poor sleep quality/unrestful sleep, delayed sleep habits) should be performed.
- Sleep–wake cycle-related habits and daily routine (fixed/regular or disordered/irregular) should be assessed, recommending the adherence to a regular life-style and constant habits.
- Meal times, amount and timing of physical activity, if any.
- The detection of excessive daytime sleepiness should elicit the hypothesis of HE and suggest the assessment of cognitive performance and ammonaemia [22].
- Detailed and complete pharmacological history should be collected, in order to identify medications that could be responsible for/contribute to sleep disturbance [68].
- Specific enquiry about sleep behaviour, with detailed assessment of sleep quality, sleep timing, and daytime sleepiness should be performed with appropriate tools, i.e. daily sleep diaries and sleep quality questionnaires [58]. These include the PSQI, to assess sleep quality and sleep disturbances over the preceding month, and to differentiate ‘good’ from ‘poor sleepers’; the simplified STSQS, which provides information on both sleep quality and sleep timing, and the ESS, to evaluate excessive daytime somnolence.

## Treatment

Since sleep–wake assessment is not part of routine hepatological practice and the pathophysiology of sleep disorders is not completely elucidated [9•], limited data are available on how sleep–wake disturbances should be treated in patients with cirrhosis. Here follows a summary of both non-pharmacological and pharmacological approaches that have been administered and evaluated over the last few years.

### Non-pharmacological Approaches

Since cirrhotic patients often suffer from ‘pill burden’, non-pharmacological treatments have often been preferred to medication [69].

For example, exposure to bright light in the early hours of the morning and avoidance of bright light exposure in the evening are sleep and light hygiene practices that should be encouraged. Drawing parallels between delayed sleep phase syndrome (DSPS), a circadian rhythm sleep disorder characterized by considerable delays in sleep onset/wake times, effectively treated with exposure to bright light in the morning [70], and the circadian deregulation and delayed melatonin response observed in cirrhosis, appropriately timed bright light therapy has been considered as a potentially beneficial non-pharmacological approach to sleep disturbances in cirrhosis [16]. One encouraging case report described in 2011 suggests that treatment with morning light might be effective [71]: an 82-year-old cirrhotic woman with a tendency to sleep–wake inversion underwent controlled lighting administration with a wall-mounted lamp, with variable light intensity/spectrum, in order to advance her sleep–wake cycle. Light was more intense and blue enriched in the morning, whereas it became less intense and red enriched during the afternoon/evening hours. As a result, a progressive improvement of sleep–wake rhythms was recorded, with reduced daytime sleepiness and fewer night awakenings. Nevertheless, a subsequent small randomized control trial of 12 cirrhotic patients did not confirm these preliminary findings, showing no obvious beneficial effect after administration of bright light therapy in terms of sleep onset, quality, and daytime sleepiness. These results are most likely in relation to the severity of disturbance at baseline, since sleep and circadian rhythms in hospitalized, decompensated patients with cirrhosis are extremely compromised [66•].

Mindfulness-based stress reduction (MBSR) is an attractive approach, the beneficial effects of which on sleep disturbances in cirrhotic patients have recently been evaluated [72]: a 4-week dedicated mindfulness and supportive group therapy approach significantly improved depression, sleep quality and H-RQoL in a group of 20 cirrhotic patients. In particular, besides a significant reduction in Beck Depression Inventory (BDI), also PSQI and, consequently, overall H-RQoL scores were significantly improved after treatment. Moreover, this approach significantly reduced the perceived burden, improved depression, and enhanced sleep quality of the caregivers of patients with cirrhosis.

Progressive neuromuscular relaxation training, lavender warm sponge bath and footbath were shown to improve sleep-related symptoms in patients with chronic liver disease, producing a significant reduction in self-rating scales of sleep [73].

### Pharmacological Treatments

Sleep disorders are often inadequately treated in cirrhosis because of the role of the liver in metabolising psychoactive medication and the fact that the therapeutic-toxic threshold of these medications is very narrow in cirrhosis [72, 74]. These patients are fairly sensitive to psychoactive medication, and the risk of precipitating severe HE when administering psychoactive drugs for sleep impairment is not negligible [9•]. For example, when an “aetiological” treatment was attempted by Spahr et al. [75] who administered the histamine H1 blocker hydroxyzine to patients with minimal HE and sleep impairment, despite an increase in sleep efficiency in treatment-group vs placebo-group, one patient developed an acute episode of HE reversible upon cessation of treatment, highlighting the need of caution when prescribing this type of drugs [75].

The impairment of melatonin metabolism observed in cirrhosis, as a result of the dysfunction of the central SCN circadian clock [26, 76], may represent an interesting field of research for future therapeutic strategies. Nevertheless, several variables need to be considered, such as overnight melatonin clearance, delays in the nocturnal rise of melatonin and in its time to peak [11, 26, 77]. Therefore, the benefits and risks of melatonin administration are worthy of specific, formal studies.

Lactulose is an effective treatment for HE. Singh et al. [78•] have recently demonstrated that improvement in HE with lactulose also leads to improvement in sleep disturbances and H-RQoL. Both sleep disorders, measured with PSQI, ESS and PSG, and H-RQoL, measured with SF-36(v2) questionnaire, have shown a significant improvement after lactulose therapy for 3 months [78•], confirming preliminary results obtained in a previous randomized controlled trial by Prasad et al. [79].

Recently, rifaximin has been shown to improve objective sleep architecture parameters on 24-h PSG, with increased REM sleep after a 28-day course of treatment, although no changes were detected in the subjective quality of sleep and sleepiness [80].

Another interesting subject of study is represented by the association of ammonia-lowering [L-ornithine-L-aspartate (LOLA)] and vigilance-enhancing medication (caffeine). A pilot study [81] conducted on six healthy volunteers and six cirrhotic patients sought to assess the effects of the administration of an amino acid challenge (AAC—see above), alone and in combination with either LOLA and caffeine. Results showed that both the administration of LOLA and caffeine could contain the post-AAC increase in capillary ammonia levels in healthy volunteers. In this group, the administration of caffeine also resulted in a reduction in subjective sleepiness, in line with previous studies [82], and in the amplitude of the EEG on several frontal/temporal-occipital sites. This was taken as evidence that the association of ammonia-lowering and vigilance-enhancing medication is worthy of further study. The timing of caffeine administration is also worthy of study in patients with cirrhosis.

Hypnotics should be used with caution, especially in decompensated patients, and chosen amongst those with negligible hepatic metabolism, short half-life, no active metabolites, and limited lipophilia, to avoid prolonged intracerebral action [9•].

A list of the available studies, evaluating specific non-pharmacological approaches or pharmacological treatments, and collective reviews, is provided in Table 2.

**Table 2:** Studies on pharmacological and non-pharmacological treatment strategies for sleep–wake disturbance in patients with cirrhosis

First author	Year	Title *Journal*	Treatment	Patients and methods	Results	Conclusions and notes
Steindl PE	1995	Disruption of the diurnal rhythm of plasma melatonin in cirrhosis *Annals of Internal Medicine*	Melatonin metabolism	7 cirrhotics and 7 controls. Neuropsychological testing to confirm subclinical HE. Plasma melatonin levels measured every 30 min for 24 h. Sleep diaries kept for 1 week before admission.	Markedly elevated melatonin levels during daytime hours in cirrhosis; significant delay in time of onset of melatonin increase and time at which melatonin levels peaked. More nocturnal awakenings and more frequent daytime naps.	Disruption of the diurnal rhythm of melatonin may reflect alterations of circadian function that could contribute to the disturbances of the sleep–wake cycle in cirrhosis.
Weyerbrock A	1996	Effects of light and chronotherapy on human circadian rhythms in delayed sleep phase syndrome: cytokines, cortisol, growth hormone, and the sleep–wake cycle *Biological Psychiatry*	Chronotherapy/Light-dark therapy	29-year-old patient with the diagnosis of DSPS: 3-day test session in the sleep laboratory, 3-week hospital chronobiological therapy, 2 nights in sleep laboratory after treatment. Interleukin-1β and γ-interferon determined in endotoxin-stimulated 48-h whole-blood cultures. Cortisol and GH plasma levels measured.	After treatment, the patient maintained a conventional sleep–wake schedule, sleep efficiency was increased and the number of wake periods was significantly reduced.	Chronotherapy effectively relieved symptoms and acceptably adjusted the sleep period. Cortisol and cytokine 24-h rhythms appear to be altered in DSPS and respond to light therapy.
Spahr L	2007	Histamine H1 blocker hydroxyzine improves sleep in patients with cirrhosis and minimal hepatic encephalopathy: a randomized controlled pilot trial *The American Journal of Gastroenterology*	Hydroxyzine	35 patients with MHE and sleep difficulties randomized to hydroxyzine 25 mg at bedtime (*N* = 17) or placebo (*N* = 18) for a 10-day period. Measurements of sleep behaviour using VAS and actigraphy, neuropsychological tests, protein s100beta serum levels, at baseline and at day 10.	Subjective improvement in sleep in 40% of hydroxyzine-treated patients but in none receiving placebo. > or = 30% increase in sleep efficiency (actigraphy) in 65% of hydroxyzine-treated patients versus 25% of patients under placebo. Acute episode of encephalopathy reversible upon cessation of hydroxyzine in one patient.	Hydroxyzine 25 mg at bedtime improved sleep behaviour in patients with cirrhosis and minimal HE. Risk of precipitating overt HE.
Prasad S	2007	Lactulose improves cognitive functions and health-related quality of life in patients with cirrhosis who have minimal hepatic encephalopathy *Hepatology*	Lactulose	Psychometry and HRQoL (SIP) of 90 cirrhotics (61 with MHE) on inclusion and 3 months later. Randomly assigned in a 1:1 ratio to receive treatment (lactulose) for 3 months (*n* = 31) or no treatment (*n* = 30).	Mean number of abnormal neuropsychological tests decreased significantly in patients in the treated group, compared with the untreated group. Mean total SIP score improved in the treated group. Improvement in HRQoL related to the improvement in psychometry.	Treatment with lactulose improves both cognitive function and HRQoL in cirrhotic patients with MHE.
Velissaris D	2008	Pituitary hormone circadian rhythm alterations in cirrhosis patients with subclinical hepatic encephalopathy *World Journal of Gastroenterology*	Melatonin and cortisol circadian patterns	26 patients with cirrhosis. 13 patients with systemic diseases not affecting the liver as controls. Neurological assessment, EEG, MRI, assays of pituitary hormone, cortisol and melatonin.	Abnormalities in pituitary hormone and melatonin circadian patterns in cirrhotic patients without OHE. Absence of cortisol secretion abnormalities in any patient, but low basal cortisol levels, correlated with EEG and brain MRI abnormalities. Melatonin identified as the only hormone associated with severity of liver insufficiency.	Abnormal pituitary hormone and melatonin circadian patterns are present in cirrhosis before the development of HE. These abnormalities may be early indicators of impending HE.
Montagnese S	2009	Sleep and circadian abnormalities in patients with cirrhosis: features of delayed sleep phase syndrome? *Metabolic Brain Disease*	Light therapy	Sleep monitored for 2 weeks, at home, with sleep diaries and actigraphy, in 35 patients with cirrhosis and 12 healthy controls; urinary aMT6s measured over 56 h, to assess circadian rhythmicity.	Later wake up and get up time and more fragmented sleep in patients. Mean 24-h urinary aMT6s outputs comparable in patients and controls but significantly lower in decompensated patients. Significant 24-h urinary aMT6s rhythms observed in 26 of 33 patients; 20 patients with normally timed urinary aMT6s peak, while delayed (> or = 06:00) in the remainder. Correlations between abnormalities in the urinary aMT6s profile and indices of sleep timing; parallel delays in sleep habits and urinary aMT6s peaks.	Delayed circadian rhythms and delayed sleep habits is reminiscent of ‘delayed sleep phase syndrome’; condition managed by attempting to resynchronise the circadian clock by exposure to bright light shortly after morning awakening.
Montagnese S	2010	On the origin and the consequences of circadian abnormalities in patients with cirrhosis *American Journal of Gastroenterology*	Circadian clock function and hepatic melatonin metabolism	20 patients with cirrhosis and 9 healthy volunteers. Plasma melatonin/cortisol concentrations measured hourly, for 24 h, in light/posture-controlled conditions. Urinary aMT6s measured simultaneously (clearance). Ability of light to suppress nocturnal melatonin synthesis assessed. Habitual sleep quality/timing evaluated using questionnaire, actigraphy, and sleep diaries.	Evidence of central circadian disruption in patients vs controls: delayed peak plasma melatonin/cortisol times and reduced plasma melatonin response to light. However, non-significant difference in mean 24 h plasma melatonin clearance between patients and volunteers. Misalignment between sleep and circadian timings in patients but no association between circadian abnormalities and impaired sleep quality.	Patients with mild to moderately decompensated cirrhosis present plasma melatonin profile abnormalities, predominantly central in origin but substantially unrelated to sleep disturbances.
De Rui M	2011	Bright times for patients with cirrhosis and delayed sleep habits: a case report on the beneficial effect of light therapy *The American Journal of Gastroenterology*	Light therapy	Sleep time/quality monitored with sleep diaries, KSS and EEG - appropriately timed light administration.	Sleep–wake rhythms progressively improved, with reduced daytime sleepiness and fewer night awakenings.	Should the observed, beneficial effect of light therapy be confirmed by formal trials, a rational, non-pharmacological, and side-effect-free treatment might become available.
De Rui M	2014	Sleep and circadian rhythms in hospitalized patients with decompensated cirrhosis: effect of light therapy *Neurochemical Research*	Light therapy	Complete sleep–wake assessment, with qualitative and semi-quantitative (actigraphic) indices of night-time sleep quality, daytime sleepiness, diurnal preference, habitual sleep timing, quality of life, mood and circadian rhythmicity (i.e. aMT6s). 5 patients randomly assigned to a single room with controlled lighting in relation to timing, spectral composition and intensity; 7 in identical rooms with standard lighting.	Sleep diaries and actigraphy: poor sleep quality, prolonged sleep latency and a reduced sleep efficiency. Quality of life globally impaired, mood moderately depressed. Serial urine collections in 7 patients: no circadian aMT6s rhythm detected in any of them, neither at baseline, nor during the course of hospitalization in either room.	Sleep and circadian rhythms in hospitalized, decompensated cirrhotic patients extremely compromised. No obvious, beneficial effects after treatment with bright light therapy, most likely in relation to the severity of disturbance at baseline.
Casula EP	2015	Acute hyperammonaemia induces a sustained decrease in vigilance, which is modulated by caffeine *Metabolic Brain Disease*	Caffeine	10 healthy volunteers, two-hourly measurements of capillary ammonia, subjective sleepiness (KSS) and vigilance (PVT), in relation to the intake of breakfast (+/−coffee), AAC and AAC + coffee.	AAC resulted in increase in: capillary ammonia levels (with highest values at approximately 4 h after the administration), subjective sleepiness ratings, PVT-based reaction times. When caffeine administered after AAC, subjective sleepiness and slowing in RTs significantly milder.	Acute hyperammonaemia induces an increase in sleepiness and decrease in vigilance, attenuated by caffeine.
Liu C	2015	Health-related management plans improve sleep disorders of patients with chronic liver disease *International Journal of Clinical and Experimental Medicine*	Lavender hot-bathing and foot-soaking, progressive relaxation	317 subjects. Initially, 197 CLD patients divided randomly into four groups for receiving lavender hot-bathing and foot-soaking, progressive relaxation, or the combination of both methods, and controls. Sleep state questionnaires to assess sleep qualities and SRSS to assess sleep disorder. Another cohort with 120 CLD patients also investigated for further confirming related findings.	The SRSS scores were significantly higher in patients with CLD than those of domestic common model and internal medicine inpatients. All three methods of intervention improved SRSS scores.	Health education could reduce risk factors and implement intervention strategies; effectively decreased occurrence of sleep disorder related symptoms.
Cassidy S	2016	Diet, Physical Activity, Sedentary Behaviour and Sleep in 1289 Adults with Liver Disease; A Cross Sectional Study of Data from the UK Biobank *Journal of Hepatology*	Diet and physical activity	Diet, physical activity, sedentary behaviour and sleep across several liver disease groups: 1. liver/biliary/pancreas disease, 2. alcoholic liver disease, 3. liver cirrhosis, compared to healthy controls.	Low physical activity, high sedentary behaviour and poor sleep as high risk characteristics of liver disease.	Lifestyle interventions should focus on targeting multiple lifestyle behaviours in the prevention and management of liver disease.
Garrido M	2016	Vigilance and wake EEG architecture in simulated hyperammonaemia: a pilot study on the effects of L-Ornithine-L-Aspartate (LOLA) and caffeine *Metabolic Brain Disease*	L-Ornithine-L-Aspartate (LOLA) and caffeine	6 cirrhotics and 6 volunteers. Hourly capillary ammonia, hourly subjective sleepiness, psychometry and wake EEG at 12:00, i.e. at the time of the maximum expected effect of the AAC.	In volunteers, post-AAC increase in capillary ammonia levels contained by both the administration of LOLA and caffeine. Reduction in subjective sleepiness and in the amplitude of the EEG after administration of caffeine. Changes in ammonia levels, subjective sleepiness and EEG less obvious in patients.	AAC-induced increase in capillary ammonia, contained by both LOLA and caffeine, especially in healthy volunteers. Caffeine also counteracted the AAC effects on sleepiness/EEG amplitude.
Singh J	2017	Sleep disturbances in patients of liver cirrhosis with minimal hepatic encephalopathy before and after lactulose therapy *Metabolic Brain Disease*	Lactulose	100 cirrhotics (n 50 MHE, n 50 no-MHE). Sleep disturbances measured with PSQI, ESS and polysomnography. HRQoL measured with SF-36(v2) questionnaire. All parameters repeated after 3 months of lactulose therapy in patients with MHE.	Poor quality of sleep and excessive daytime sleepiness more common in patients with MHE, compared to those without MHE. With lactulose therapy, improvement in MHE in 21 patients (arterial ammonia levels, CFF, PHES, PSQI, ESS, HRQoL).	Excessive daytime sleepiness and impaired sleep quality in patients with MHE correlate with neuropsychiatric impairment. Improvement in MHE with lactulose also leads to improvement in sleep disturbances and HRQoL.
Bajaj JS	2017	Mindfulness-based stress reduction therapy improves patient and caregiver-reported outcomes in cirrhosis *Clinical and Translational Gastroenterology*	Mindfulness-based stress reduction therapy	20 patient/caregiver dyads included. Cirrhotic outpatients with mild depression on screening with an adult caregiver enrolled. At baseline, BDI, sleep (PSQI, ESS), anxiety and HRQoL for both patients/caregivers; caregiver burden and patient CHE status measured. Structured MBSR program with four weekly hour-long group sessions interspersed with home practice using CDs.	Significant improvement in BDI, PSQI and overall HRQoL but not in anxiety or CHE rates in patients. Similarly caregiver burden and depression reduced while caregiver sleep quality improved.	A short program of mindfulness and supportive group therapy significantly improves PRO and caregiver burden in patients with depression. This non-pharmacological method could alleviate psychosocial stress in patients with end-stage liver disease and their caregivers.
Bruyneel M	2017	Improvement of sleep architecture parameters in cirrhotic patients with recurrent hepatic encephalopathy with the use of rifaximin *European Journal of Gastroenterology and Hepatology*	Rifaximin	15 patients. 24-h polysomnography and 7-day actigraphy. REM sleep: indicator of good sleep quality. Questionnaires assessing the quality of sleep and sleepiness. The same assessment repeated after a 28-day course of rifaximin	Polysomnography: long TST and limited REM sleep. Actigraphy: impaired number of steps. Questionnaires: impaired sleep quality and excessive daytime sleepiness. After rifaximin, decrease of HE scores, increase of REM sleep, no changes for TST, number of steps, and on questionnaires.	Rifaximin improves objective sleep architecture parameters on polysomnography, with no changes in the subjective quality of sleep and sleepiness.

*AAC*, amino acid challenge; *aMT6s*, 6-sulphatoxymelatonin; *BDI*, Beck Depression Inventory; *CFF*, critical flicker frequency; *CLD*, chronic liver disease; *DSPS*, delayed sleep phase syndrome; *EEG*, electroencephalogram; *ESS*, Epworth Sleepiness Scale; *GH*, growth hormone; *HE*, hepatic encephalopathy; *MHE/CHE*, minimal/covert hepatic encephalopathy; *OHE*, overt hepatic encephalopathy; *HRQoL*, health-related quality of life; *KSS*, Karolinska Sleepiness Scale; *MBSR*, mindfulness-based stress reduction; *MCTQ*, Munich ChronoType Questionnaire; *MRI*, magnetic resonance imaging; *NAFLD*, non-alcoholic fatty liver disease; *NPV*, negative predictive value; *PFC*, plasma free cortisol; *PRO*, patient-reported outcomes; *PSQI*, Pittsburgh Sleep Quality Index; *PSS*, porto-systemic shunt; *PVT*, psychomotor vigilance task; *REM*, rapid eye movement; *RTs*, reaction times; *SIP*, sickness impact profile; *SRSS*, self-rating scores of sleep; *STSQS*, sleep timing and sleep quality screening; *TST*, total sleep time; *VAS*, Visual Analogue Scale

## Future Perspectives

In conclusion, over the last few years, progress has been made in terms of both the description of the sleep–wake abnormalities associated with cirrhosis, and the understanding of their pathophysiology. However, this has not yet translated into well-defined therapeutic strategies or well-designed treatment trials.

In parallel, a phenomenal amount of progress has been made by basic chronobiologists in the understanding of the functions of the liver clock and its role, dependence and ability to dissociate from the main, cerebral circadian clock [83, 84]. Meanwhile, the circadian and sleep–wake consequences of the timing of meals [85] and physical activity [86] are being better defined.

The 2017 Nobel prize in Physiology/Medicine went to the discovery of the molecular mechanisms controlling circadian rhythms. Evidence of the health consequences of both circadian deregulation and sleep loss [87, 88] is emerging at an impressive rate. Such exciting discoveries have remarkable translational, clinical and therapeutic potential, and hepatology may represent a favourable environment in this respect. We should attempt to rise to the challenge.
