# Correction: Assessment and Management of Sleep Disturbance in Cirrhosis

**DOI:** 10.1007/s11901-018-0402-1

**Published:** 2018-05-23

**Authors:** Chiara Formentin, Maria Garrido, Sara Montagnese

**Affiliations:** 1 Department of Medicine, University of Padua, Via Giustiniani, 2, 35128 Padua, Italy; 2 Department of Physiology, Neuroimmunophysiology and Chrononutrition Research Group, Faculty of Science, Avda. Elvas s/n, 06006 Badajoz, Spain

**Correction: Current Hepatology Reports (2018) 17(1):52–69**

10.1007/s11901-018-0390-1

The article Assessment and Management of Sleep Disturbance in Cirrhosis, written by Chiara Formentin, Maria Garrido, and Sara Montagnese, was originally published electronically on the publisher’s internet portal (currently SpringerLink) on 13 February 2018 without open access. With the author(s)’ decision to opt for Open Choice the copyright of the article changed on May 2018 to © The Author(s) 2018 and the article is forthwith distributed under the terms of the Creative Commons Attribution 4.0 International License (http://creativecommons.org/licenses/by/4.0/), which permits use, duplication, adaptation, distribution and reproduction in any medium or format, as long as you give appropriate credit to the original author(s) and the source, provide a link to the Creative Commons license and indicate if changes were made.

**Open Access** This article is distributed under the terms of the Creative Commons Attribution 4.0 International License (http://creativecommons.org/licenses/by/4.0/), which permits use, duplication, adaptation, distribution and reproduction in any medium or format, as long as you give appropriate credit to the original author(s) and the source, provide a link to the Creative Commons license and indicate if changes were made.

The original article has been corrected.

